# Treatment of myocardial interstitial fibrosis in pathological myocardial hypertrophy

**DOI:** 10.3389/fphar.2022.1004181

**Published:** 2022-09-30

**Authors:** Fuyu Zhu, Peng Li, Yanhui Sheng

**Affiliations:** ^1^ Department of Cardiology, The Affiliated Suzhou Hospital of Nanjing Medical University, Suzhou Municipal Hospital, Gusu School, Nanjing Medical University, Suzhou, China; ^2^ Department of Cardiology, the First Affiliated Hospital of Nanjing Medical University, Nanjing, China; ^3^ Department of Cardiology, Jiangsu Province Hospital, Nanjing, China

**Keywords:** pathological myocardial hypertrophy, myocardial interstitial fibrosis, myofibroblasts, anti-fibrosis, biomarkers

## Abstract

Pathological myocardial hypertrophy can be caused by a variety of diseases, mainly accompanied by myocardial interstitial fibrosis (MIF), which is a diffuse and patchy process, appearing as a combination of interstitial micro-scars and perivascular collagen fiber deposition. Different stimuli may trigger MIF without cell death by activating a variety of fibrotic signaling pathways in mesenchymal cells. This manuscript summarizes the current knowledge about the mechanism and harmful outcomes of MIF in pathological myocardial hypertrophy, discusses the circulating and imaging biomarkers that can be used to identify this lesion, and reviews the currently available and potential future treatments that allow the individualized management of patients with pathological myocardial hypertrophy.

## 1 Introduction

Myocardial hypertrophy can be broadly defined as the increase of heart mass (Bernardo et al., 2010). The main function of the heart is to maintain the perfusion of peripheral organs to meet the needs of normal and stress conditions. However, the growth of postnatal heart is affected by its functional load. In order to accomplish this task with increased preload or afterload, the size of cardiomyocytes typically increases, which is called hypertrophy. Hypertrophy can be divided into physiological hypertrophy and pathological hypertrophy. Both types of hypertrophy involve the enlargement of single cardiomyocytes, which were initially developed as an adaptive response to cardiac stress, while they remarkably differ in potential molecular mechanisms, cardiac phenotypes, and prognosis (Nakamura and Sadoshima, 2018). Physiological hypertrophy is mainly caused by normal postpartum growth, pregnancy, and exercise (Perrino et al., 2006). Pathological hypertrophy is caused by chronic hypertension, aortic stenosis, mitral or aortic regurgitation, myocardial infarction, storage diseases (e.g., lipid, glycogen and misfolded protein storage diseases), and hereditary cardiomyopathy (e.g., hypertrophic cardiomyopathy) caused by gene mutations encoding sarcomere protein (Devereux et al., 2000; Turkbey et al., 2010). Over time, physiological hypertrophy can maintain cardiac function, while pathological hypertrophy is highly accompanied by cardiomyocyte death, leading to interstitial and perivascular fibrosis and adverse cardiovascular events, including heart failure (HF), arrhythmia, and death.

At the cellular level, pathological hypertrophy is caused by cardiomyocyte hypertrophy, expansion of surrounding stroma, and loss of extracellular homogeneity (Rodrigues et al., 2016). As the key component of pathological hypertrophy, interstitial expansion is the result of the joint action of extracellular edema and interstitial fibrosis, which is mainly secondary to the aggregation of mature cross-linked collagen fibers (Halliday and Prasad, 2019). The diffuse and unbalanced accumulation of collagen in myocardial stroma is called myocardial interstitial fibrosis (MIF) (González et al., 2018). MIF is a diffuse and patchy process. It is a histological marker of several heart diseases that change myocardial structure and function, and can be associated with the progression of HF. The main manifestations of MIF were interstitial micro-scar formation, perivascular collagen fiber deposition, and increased thickness of tendon collagen chain (Díez et al., 2020). Although a large number of research evidences have explained the mechanism of MIF in pathological myocardial hypertrophy, it has not yet fully realized the transformation of these knowledge into a practical and effective treatment of pathological myocardial hypertrophy. This review will focus on the currently available and potential future treatment methods of MIF in pathological myocardial hypertrophy caused by hypertensive heart disease (HHD), aortic stenosis, diabetic cardiomyopathy, hypertrophic cardiomyopathy and so on.

### 1.1 Classification and characteristics of myocardial interstitial fibrosis

Myocardial fibrosis is mainly divided into replacement fibrosis and interstitial fibrosis. Replacement fibrosis occurs in myocardial injury (i.e., myocardial infarction), which is characterized by excessive collagen deposition and micro-scar formation. The reason is that after myocardial cells are damaged, they are replaced by activated fibroblasts to form the main scar containing type I collagen (de Jong et al., 2011). MIF can be further divided into reactive MIF and invasive MIF (Herum et al., 2017). Reactive MIF is the accumulation of collagen in the extracellular space due to the imbalance of collagen metabolism without cell death (Liu et al., 2021). It may be manifested as a thick fibrotic sheath located around the intramural coronary artery and arterioles, or around the cardiac muscle bundle (i.e., perimuscle) and a single cardiomyocyte (i.e., intima) (Halliday and Prasad, 2019). Invasive MIF occurs in patients with Fabry disease which is a rare genetic disease characterized by dysfunction of sphingolipid function and catabolism (Disertori et al., 2017). The potential reason why MIF does not lead to cardiomyocyte death may be that MIF is often secondary to some harmful stimuli such as pressure overload or myocardial inflammation, which do not lead to cardiomyocyte death. Replacement fibrosis and reactive interstitial fibrosis coexist in the majority of patients. It is essential to clarify whether these two histological types of fibrosis represent different entities or whether they represent different stages of development of the same disease (Halliday et al., 2017). For instance, the latest research on patients with hypertrophic cardiomyopathy showed that fibrosis is initially characterized by perivascular, perisomatic and intramuscular deposits, while as the disease progresses, fibrosis is mainly characterized by alternative scars (Galati et al., 2016).

MIF is a scar event in myocardium, which is associated with myocardial hypertrophy. It occurs in almost all types of heart disease and has different manifestations (Weber et al., 2013; Segura et al., 2014). For example, MIF in HHD is composed of fine connective tissue and mainly manifested as interfiber fibrosis, while the MIF of hypertrophic cardiomyopathy is composed of coarse collagen fibers and mainly manifested as plexiform fibrosis (Sugihara et al., 1988). Collagen deposition is the leading feature of MIF, especially type I and type III collagen, as well as (Liu et al., 2021) type I and type III collagen fibers are the main structural proteins forming fibrous tissue (Whittaker, 1995; Fomovsky et al., 2012). Although MIF is patchy, the area of fibrosis increases from the outer to the inner third of the ventricular free wall in some patients with heart diseases, such as HHD, aortic stenosis, and hypertrophic cardiomyopathy (Treibel et al., 2018). This may be related to transmural pressure, wall stress, and changes of coronary microcirculation, leading to relative endocardial ischemia (Díez et al., 2020). An evidence showed that MIF is not only related to the scope of fiber deposition, but also related to the collagen composition and physicochemical properties of fibers. For instance, in pathological myocardial hypertrophy caused by hypertension or aortic stenosis, the content of type I collagen is higher than that of type III collagen due to the excessive accumulation of type I collagen fibers (López et al., 2014; Echegaray et al., 2017). However, in diabetic cardiomyopathy, the content of type III collagen is higher than that of type I collagen (Shimizu et al., 1993). In hypertrophic cardiomyopathy, there is no difference between the content of the two types of collagen (Boerrigter et al., 1998). Collagen fibers have physical and chemical properties such as insolubility, degradation resistance and hardness. The insolubility, degradation resistance and stiffness of collagen fibers depend on the degree of intermolecular covalent bonding (i.e., cross-linking) between their constituent fibers (Díez et al., 2020). Type I collagen fibers are mainly related to the coarse fibers that give tensile strength, while type III collagen fibers usually form fine fibers and maintain the elasticity of the matrix network (Kong et al., 2014). Due to type I collagen fibers are heavily cross-linked, the stiffness of type I collagen fibers in human myocardium is higher than that of type III collagen fibers. Some studies have proved through Young’s modulus evaluation that there is a direct correlation between the ratio of collagen type I to collagen type III and myocardial stiffness in HF patients caused by severe aortic stenosis (López et al., 2021). However, it is still unclear how the ratio of type I collagen to type III collagen affects the specific pathological phenotype of MIF. Another feature of MIF is that collagen with cross-linking density matures and then forms fibrotic scars. This feature may increase the tensile strength of the scar, affect the degree of cardiac relaxation and contractility, and limit cardiac function (La et al., 2017).

## 2 Mechanism of myocardial interstitial fibrosis

**FIGURE 1:**
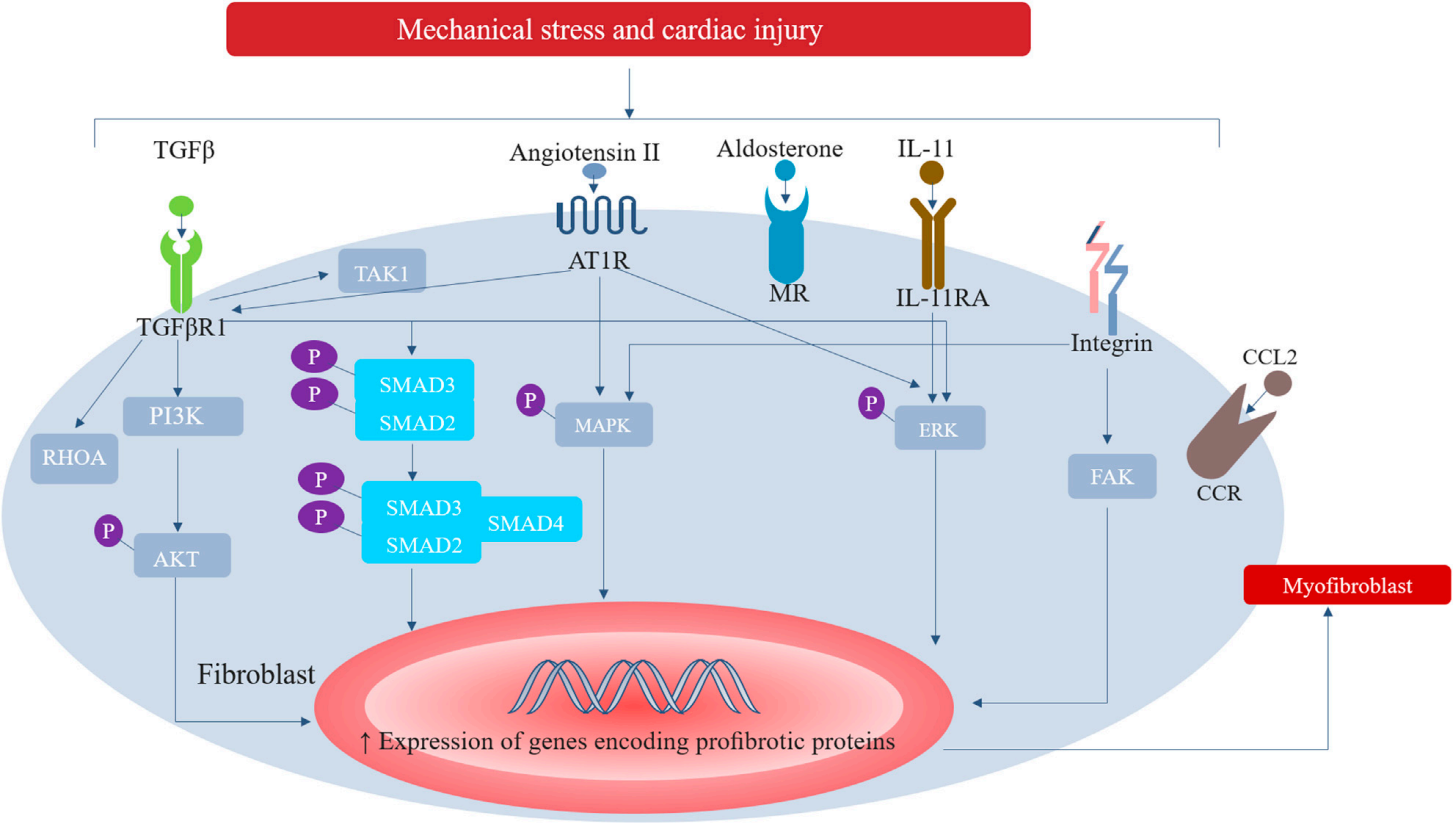
Major signalling pathways involved in the activation of cardiac fibroblasts. In response to increased mechanical stress and to cardiac injury, inflammatory signalling cascades (triggered by cytokines and chemokines), mechanosensitive mechanisms (mediated by integrins and ion channels), and neurohumoral pathways (such as renin-angiotensin-aldosterone system) can activate fibroblasts into extracellular matrix-synthesizing myofibroblasts. Among them, TGF-β is the main regulator of fibrogenesis through SMAD-dependent pathway and none-canonical signal cascade. Ang II also plays an important role in cardiac fibrosis, which activates profibrotic cascades, such as TGF- β/SMAD, ERK1/2, AKT and MAPK. AT1R, type 1 angiotensin II receptor; CCL2, CC- chemokine ligand 2; CCR, CC- chemokine receptor; ERK, extracellular-signal- regulated kinase; FAK, focal adhesion kinase; IL-11RA, IL-11 receptor subunit- α; MAPK, mitogen-activated protein kinase; MR, mineralocorticoid receptor; PI3K, phosphoinositide 3-kinase; RHOA, transforming protein RHOA; TAK1, TGFβ-activated kinase 1; TGFβR1, TGFβ receptor type 1.

Myocardial fibrosis is secondary to various stimuli, including ischemia, poisoning, metabolism, infection, genetics, and hemodynamics. These stimuli directly activate the fibrotic pathway or lead to cardiomyocyte dysfunction (González et al., 2018). In reactive MIF, without cell death, a variety of fibrotic signaling pathways in mesenchymal cells (e.g., fibroblasts) may be activated by different stimuli to cause fibrosis, including the following stimuli: mechanical stress associated with pressure overload of HHD and aortic stenosis, defects caused by various causal mutations in sarcomere structure and function of hypertrophic cardiomyopathy, metabolic damage associated with hyperglycemia in diabetic cardiomyopathy, or coronary microvascular endothelial damage in HF with preserved ejection fraction (HFpEF) (Díez et al., 2020). Fibroblasts undergo a response known as “activation”, which is characterized by a transition of quiescent cells into myofibroblast-like phenotype, while some other cells, including macrophages, cardiomyocytes, and endothelial cells, indirectly participate in the fibrotic response by secreting fibrogenic mediators (Kong et al., 2014). Fibroblasts can sense damage-related molecular patterns (DAMP), which lead to activate pro-inflammatory responses. Molecular patterns associated with injury include proinflammatory cytokines (i.e., interleukin-1α), intracellular molecules released by dead cells (i.e., heat shock proteins), extracellular matrix (ECM) molecules up-regulated by injury (i.e., fibronectin), or molecules modified by a pathological environment (i.e., advanced glycation end products (AGEs)). Fibroblasts also provide membrane receptors for many growth factors, cytokines, and neurohumoral factors that regulate signaling pathways, including platelet-derived growth factor (PDGF), angiotensin-II (especially type 1 receptor), connective tissue growth factor (CTGF), and transforming growth factor- *ß* (TGF-β). In injured myocardium caused by a variety of physical and chemical factors, cardiomyocytes, macrophages, and endothelial cells can release cytokines, chemokines, and growth factors (e.g., transforming growth factor-β (TGF-β)). These factors work together with mechanical stress and neurohormone activation to make fibroblasts proliferate and differentiate into myofibroblasts through a variety of signal pathways. These signaling pathways include DAMP-dependent pathways, inflammatory signalling cascades (triggered by cytokines and chemokines), mechanosensitive mechanisms (mediated by integrins and ion channels), and neurohumoral pathways (such as renin-angiotensin-aldosterone system). Among them, TGF-β is the main regulator of fibrogenesis through SMAD-dependent pathway and none-canonical signal cascade. Ang II also plays an important role in cardiac fibrosis, which activates profibrotic cascades, such as TGF-β/SMAD, ERK1/2 (extracellular-signal- regulated kinase1/2), AKT (protein kinase B) and MAPK (mitogen-activated protein kinase) signal transduction (Subbaiah et al., 2022). The pathophysiological role of Ang II can reconnect the transcriptome of fibroblasts to promote the activation of fibroblasts with enhanced their ability to migrate, proliferate, secrete proinflammatory mediators, and produce ECM proteins (Subbaiah et al., 2022) (Figure 1). Additionally, myofibroblasts can also be transformed from circulating fibroblast precursor cells, epicardial epithelial cells, and endothelial cells (Gyöngyösi et al., 2017).

**FIGURE 2:**
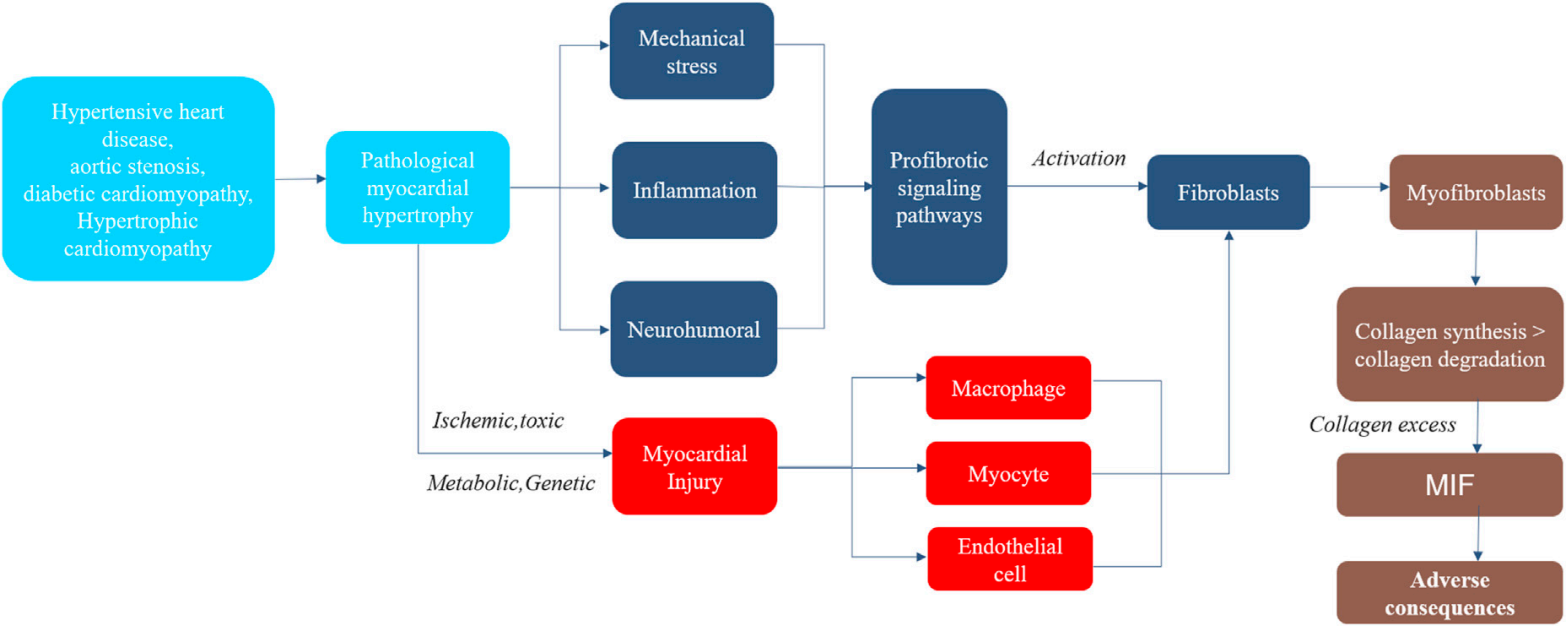
The Process of Myocardial Interstitial Fibrosis in pathological myocardial hypertrophy. Steps in the process of myocardial interstitial fibrosis, including major mechanisms and consequences. MIF, myocardial interstitial fibrosis.

Myofibroblasts are the main mediating cells in MIF, possessing the characteristics of proliferation and secretion. They regulate cytokine secretion and collagen synthesis, promote the formation of non-functional scar, and contribute to the renewal of extracellular matrix and collagen deposition (Liu et al., 2021). Myofibroblasts possess the characteristics of synthetic active fibroblasts because they have the combined ultrastructural and phenotypic features of smooth muscle cells obtained through the formation of contractile stress fibers and *de novo* expression of *a*-smooth muscle actin, as well as extensive endoplasmic reticulum (Weber et al., 2013). The fiber secretory group of myofibroblasts is composed of molecules required to change the collagen turnover of extracellular fibrils and promote MIF, as well as autocrine and paracrine factors that further simulate their proliferative and metabolic activities, so as to perpetuate the fiber formation in the damaged myocardium, thereby maintaining fiber formation (Bomb et al., 2016). The most important proteins secreted by myofibroblasts are type I and type III procollagen precursors and enzymes that directly interfere with the extracellular synthesis, deposition, and degradation of type I and type III collagen fibers, such as type I procollagen amino terminal protease (disintegrin and matrix metalloproteinase (MMP)) and type I procollagen carboxyl terminal protease, or procollagen C-proteinase (PCP, also known as bone morphogenetic protein-1) (Ricard-Blum, 2011). These enzymes convert terminal propeptides of the procollagen precursors secreted by myofibroblasts into mature collagen fibroin molecules by lysing them. Subsequently, lysyl oxidase (LOX) family plays a catalytic role in formation of the final collagen fibers that may deposit in the myocardium through covalent bonds (i.e., cross-linking) between adjacent fiber polypeptide chains (Díez et al., 2020). The increase of mature fibers destroys the balance between fiber production and degradation and extracellular expansion, that is, fiber formation and deposition are dominant, rather than its degradation and removal (Bishop and Laurent, 1995). The diffuse and unbalanced aggregation of final mature fibers in myocardial stroma leads to MIF (Figure 2).

## 3 Adverse consequences of myocardial interstitial fibrosis

**FIGURE 3:**
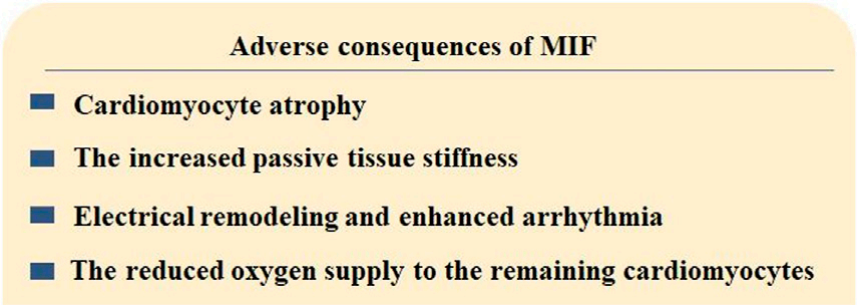
Adverse consequences of MIF. MIF, myocardial interstitial fibrosis.

MIF is associated with the adverse effects of myocardial function and can lead to a variety of adverse consequences, including cardiomyocyte atrophy, increased passive tissue stiffness, electrical remodeling and enhanced arrhythmia, and reduced oxygen supply to the remaining cardiomyocytes (Weber et al., 2013). These adverse consequences may lead to the left ventricular (LV) dysfunction, arrhythmia, and other disorders, affecting the prognosis of cardiac function in patients with pathological myocardial hypertrophy, which indicates uncontrolled and progressive accumulation of fibrotic tissue (Figure 3).

### 3.1 Cardiomyocyte atrophy

Fibrosis was reported as a “key determinant” of myocardial heterogeneity in heart diseases (Weber et al., 2013). Although the size of cardiomyocytes in the myocardium is mainly variable, (Campbell et al., 1989), this heterogeneity is more prominent in the diseased heart, because fibrous collagen tendrils produced by scar tissue trap adjacent cardiomyocytes, reducing the workload of cardiomyocytes, thereby inducing cardiomyocyte atrophy (Fidziańska et al., 2010). For instance, atrophic, while viable cardiomyocytes trapped by fibrillary collagen at scar and perivascular fibrotic sites are anatomically separated from cardiomyocytes with smaller remodeling sites found in pressure-overload hypertrophic hearts. Re-expression of the expressed *ß*-myosin heavy chain (β-MHC) gene is a well-documented marker of pathological cardiac hypertrophy (Pandya et al., 2006; López et al., 2011).

### 3.2 The increased passive tissue stiffness

The increase of collagen cross-linking may lead to the hardening of fibrous tissue, and the stiffness of type I collagen fiber is higher than that of type III collagen fiber. Therefore, the passive tissue stiffness increased with the increase of the deposition of rigid and highly cross-linked type I collagen fibers (López et al., 2012). At the same time, hardened fibrous tissue can also further promotes fibrosis *via* mechanosensitive pathways and *via* liberation of latent TGF-β.With the increase of myocardial stiffness caused by collagen accumulation, diastolic dysfunction may occur (Ohsato et al., 1992; Díez et al., 2002). A previous study showed that MIF is associated with the LV stiffness and diastolic dysfunction in patients with HHD, aortic stenosis, hypertrophic cardiomyopathy, and HFpEF through histological evaluation or CMR imaging (Maragiannis et al., 2018).

### 3.3 Electrical remodeling and enhanced arrhythmia

The electrical remodeling and enhanced arrhythmia caused by MIF are due to the accumulation of matrix protein and the existence of myofibroblasts. The pathophysiological mechanism is mediated by intracellular Ca^2+^-overload and oxidative stress (Hothi et al., 2008). A previous study demonstrated that MIF impairs myocardial electrophysiology by slowing down propagation of action potential, initiating reentry, promoting posterior depolarization, and increasing ectopic autonomy, which together lead to an increased risk of ventricular arrhythmia (Nguyen et al., 2017). For instance, in patients with hypertrophic cardiomyopathy, MIF has a certain influence on the electrophysiological changes that mediate ventricular arrhythmia (Kawara et al., 2001). Ventricular arrhythmia is affected by the degree of MIF, and is not correlated with other confounding factors, including the LV dysfunction. Moreover, it has been reported that the relationship between MIF and ventricular arrhythmia has no dependency on LV function in patients with HHD (McLenachan and Dargie, 1990). MIF has been recognized as a risk factor for sudden cardiac death in patients with HHD, hypertrophic cardiomyopathy, and non-ischemic dilated cardiomyopathy (Bockstall and Link, 2012; Gulati et al., 2013; Shenasa and Shenasa, 2017).

### 3.4 The reduced oxygen supply to the remaining cardiomyocytes

The heart is a specialized aerobic organ. Its long-term performance requires a stable oxygen supply to maintain the energy demand. The coronary vasodilatory reserve is mainly used to ensure the delivery of sufficient oxygen to meet the oxygen demand of cardiomyocytes. Therefore, the myocardium can adapt to significant changes in oxygen demand beyond its aerobic threshold and before beginning anaerobic metabolism (Weber et al., 1980). Perivascular fibrosis of intramural coronary arteries and arterioles, as well as the presence of myofibroblasts, can lead to coronary lumen stenosis and threaten the survival of cardiomyocytes (Warnes et al., 1984; Olivotto et al., 2011). In patients with HHD, hypertrophic cardiomyopathy or diabetes cardiomyopathy, the severity of MIF is related to the severity of coronary microvascular disease, including the anatomical abnormality of vascular wall and the rarefaction of capillaries (Mohammed et al., 2015).

As for whether MIF is a direct harmful cause of cardiac function, studies have evaluated collagen deposition and/or collagen cross-linking through histological analysis, proving that there is a direct correlation between MIF severity and diastolic dysfunction in patients with HF, which supports the direct involvement of MIF in LV diastolic dysfunction (Izawa et al., 2005). However, there are differences in this regard, because several articles have proved that fibrosis itself does not cause electrical problems or arrhythmias.

## 4 Diagnosis of myocardial interstitial fibrosis

Because MIF affects the clinical outcome of patients with pathological myocardial hypertrophy, MIF may need to be used as an evaluation indicator of clinical treatment for these patients. Endomyocardial biopsy has higher accuracy in characterizing interstitial changes and can specify their components, while it cannot be used routinely and continuously because of its limitations, such as invasiveness, complications, and sampling errors (Halliday and Prasad, 2019). Therefore, a routine practice requires alternative non-invasive methods, such as cardiovascular magnetic resonance (CMR) imaging and circulating biomarkers.

CMR imaging-derived parameters are commonly used to evaluate myocardial fibrosis, including late gadolinium enhancement (LGE) and T1 mapping, which can be divided into natural T1, post contrast T1, and extracellular volume fraction (ECV). LGE can be utilized to identify focal collagen deposition in scars after large-area infarction, while T1 mapping can be used to identify diffuse collagen deposition in MIF (Díez et al., 2020). Clinical studies have demonstrated that ECV and LGE can evaluate the degree of diffuse collagen deposition by collagen volume fraction, and ECV is superior to LGE (Diao et al., 2016). On the other hand, neither LGE nor ECV can qualitatively judge the composition and molecular organization of collagen fibers in MIF (Messroghli et al., 2017). Therefore, due to this limitation of CMR-derived biomarkers, another method based on circulating biomarkers may be needed to quantitatively and qualitatively judge MIF.

Of the several proposed MIF biomarkers, only a limited number of them are associated with histologically confirmed MIF, such as serum carboxy terminal propeptide of type I procollagen (PICP), serum amino terminal propeptide of type III procollagen (PIIINP), and the ratio of serum type I collagen terminal peptide to serum matrix metalloproteinase 1 (López et al., 2015). PICP is formed during the extracellular conversion of type I procollagen to type I collagen by PCP enzyme. It was found that the PICP level was closely associated with the deposition of type I collagen in myocardium of patients with HHD and HF (López et al., 2012). PIIINP is produced during the extracellular transformation of type III procollagen into type III collagen by procollagen amino terminal protease. It was also found that its level is highly correlated with the degree of myocardial type III collagen deposition in patients with ischemic heart disease or idiopathic dilated cardiomyopathy and HF (Klappacher et al., 1995). Similarly, there are some limitations in the detection of MIF biomarkers. For instance, they do not have heart specificity, and the change of their concentration may represent the comprehensive abnormality of cardiovascular collagen or the influence of complications affecting collagen metabolism.

## 5 Treatment of myocardial interstitial fibrosis

Existing clinical evidence shows that MIF may affect the response of patients with pathological myocardial hypertrophy to treatment, and there is no routine use of specific anti fibrosis drugs in clinical practice. Therefore, for patients with pathological myocardial hypertrophy, anti-fibrosis and cardiac protection strategies need to be developed, which are mainly introduced from the following three aspects. But cardiac fibrosis is an important part of the pathophysiological response to injury. Sometimes appropriate fibrosis has a protective effect on the heart, so different pathological backgrounds of fibrosis need different treatments.

### 5.1 Drugs with some exhibited clinical benefit

The clinical studies have shown that MIF is a target of drugs interfering with the renin-angiotensin- aldosterone axis. A large number of drugs were found to play an anti-MIF role by acting on this axis. For instance, in the treatment of patients with HHD, the use of angiotensin receptor blocker losartan or angiotensin-converting enzyme inhibitor lisinopril can reduce the degree of fibrosis deposition and LV stiffness, and improve LV diastolic dysfunction accordingly (Tacke and Trautwein, 2015). Similarly, mineralocorticoid receptor antagonist spironolactone can also reduce collagen deposition and LV stiffness in patients with HF and improve diastolic dysfunction (Izawa et al., 2005). These drugs have been reported to have anti-MIF efficacy and safety, but there is still a residual burden of fibrosis. Therefore, new treatment methods are urgently required for more effective anti-fibrosis treatment. For instance, torasemide, a new loop diuretic, may directly target MIF. Studies have demonstrated that the use of torasemide in patients with HHD can affect the degree of collagen deposition, reduce PCP activation and LOX expression (López et al., 2009). However, this was obtained in the study of a small number of patients receiving short-term treatment. Rigorous and large-scale clinical studies are still needed to prove whether torasemide targets MIF in a clinically effective manner (Díez et al., 2020).

### 5.2 Drugs that have shown promise but only in pre-clinical models

On the other hand, data obtained from experimental studies revealed that some drugs with proven safety that have been used in clinical practice may treat MIF in pathological myocardial hypertrophy through new mechanisms (Johnson et al., 2017). For instance, in HF mice with cardiac pressure overload and diabetes, the combined use of enkephalinase inhibitors and angiotensin receptor blockers, sacubitril/valsartan (a new class of drugs called angiotensin receptor neprilysin inhibitors) relieved MIF and improved LV function (Burke et al., 2019). The antifibrotic effect of sacubitril/valsartan may be due to the specific inhibition of neutral lysozyme beyond the effect of angiotensin receptor blockers. The mineralocorticoid receptor antagonist eplerenone can prevent increased collagen deposition and left ventricular systolic dysfunction in pressure overloaded mice (Franco et al., 2006). In addition, riociguat, the soluble guanylate cyclase stimulator, can reduce collagen deposition in HF mice caused by transverse aortic coarctation (Pradhan et al., 2016).

Studies have shown that sodium glucose cotransporter 2 (SGLT2) inhibitors, used as hypoglycemic drugs in patients with diabetes, also have anti MIF effects and can reduce the risk of cardiovascular death in patients with pathological myocardial hypertrophy. Such as, empagliflozin can attenuate MIF and improve diastolic dysfunction in diabetic mice (Habibi et al., 2017). Some studies have also shown that in pressure overloaded mice caused by transverse aortic coarctation, dapagliflozin treatment can reduce collagen deposition and improve left ventricular function (Shi et al., 2019). However, because SGLT2 is not expressed in the heart and its antifibrosis effect is not related to metabolic and/or hemodynamic changes, some people believe that it may reflect the direct pleiotropic effect of drugs on myocardium (Packer et al., 2017). Therefore, it remains to be tested whether SGLT2 inhibitors can effectively reduce MIF.

Celecoxib, a selective cyclooxygenase-2 (COX-2) inhibitor, has been shown in a study to reduce pathological cardiac hypertrophy and fibrosis (Zhao et al., 2021). A number of evidences show that COX-2 is related to cardiac hypertrophy and fibrosis. Specific overexpression of COX2 promotes cardiac hypertrophy, while COX-2 inhibition can improve the hypertrophy and fibrosis induced by angiotensin and aldosterone (Wang et al., 2010). This study used cryoinjury (CI) to establish a mouse model of cardiac hypertrophy and fibrosis. It was found that celecoxib inhibited the production of proinflammatory cytokines and the expression of adhesion molecule genes, increased the recruitment of M1 like macrophages, and reduced cardiac hypertrophy and fibrosis (Zhao et al., 2021). However, the regulatory mechanism of COX-2 in immune-mediated cardiac hypertrophy and fibrosis is not completely clear.

Platycodin D (PD) is a triterpenoid saponin isolated from the widely used traditional Chinese medicine extracted from the root of Platycodon grandiflorum (PG), which has anticancer and antiangiogenic effects (Lee et al., 2015). Studies have shown that PD can reduce the incidence rate and mortality of cardiovascular disease by inhibiting cardiac fibrosis and hypertrophy (Lin et al., 2018). PD can reduce the accumulation of collagen in cardiac hypertrophy rats caused by hypertension, possibly by reducing the expression of MMP2, MMP9 and TGF- *ß* 1 to inhibit cardiac hypertrophy and fibrosis. In addition, in preclinical animal models, PD treatment did not show related mortality at up to 2000 mg/kg, indicating that PD has great potential for clinical use because it has no obvious toxicological signs (Lee et al., 2011).

It has also been reported that the liver X receptor (LXR) agonist AZ876 can prevent pathological cardiac hypertrophy and fibrosis in a study (Cannon et al., 2015). This study has shown that AZ876 treatment can inhibit the induction of profibrotic gene expression and reduce MIF in a mouse model of pathological cardiac hypertrophy caused by chronic pressure overload. Its potential mechanism may be related to the weakening of TGF-β - Smad2/3 signal transduction. Two major cell types in the cardiac, cardiomyocytes and fibroblasts, express LXR. These two kinds of cells are the direct targets of AZ876 mediated cell protection from hypertrophy and fibrosis (Cannon et al., 2015). AZ876 activation can prevent TGF-β and Ang II induced collagen synthesis and myofibroblast differentiation. AZ876 has dual effects of anti-myocardial hypertrophy and anti-fibrosis, and can also reduce atherosclerosis without affecting the liver or plasma triglyceride levels (van der Hoorn et al., 2011). However, the cardiac protective potential of AZ876 remains to be further explored.

Finally, pirfenidone and tranilast, two inhibitors of TGF-β signaling, have also been shown to have anti-fibrotic effects in several MIF models (Edgley et al., 2012). Because the long-term use of either of these two drugs may produce hepatotoxicity and lead to liver failure, further experiments are needed to alleviate MIF *via* studies that support safely and effectively targeting of TGF-β signaling (Fang et al., 2017).

### 5.3 Other potential treatments

The clinical principle of anti-MIF strategies mainly aims to reduce the excessive deposition of fibrous tissue, which may be achieved through the inactivation of inflammatory pathways and the establishment of anti-inflammatory microenvironment, or the inactivation and elimination of myofibroblasts, or the excessive synthesis and deposition of collagen (Díez et al., 2020). Based on this principle, we can find new anti-MIF markers in pathological myocardial hypertrophy, such as non-coding RNAs (ncRNAs), epigenetic modifiers, Endoplasmic reticulum (ER),mitochondrion and so on, which can be used as new molecular drug targets (Park et al., 2019). However, these new potential anti MIF strategies are still not really used in clinical treatment, and more research is needed to clarify.

NcRNAs play a key role in DNA replication, transcription and post transcriptional gene expression, chromatin processing, and maintaining mRNA stability and genomic integrity (Yang et al., 2016). In terms of pathological cardiac hypertrophy and MIF, microRNA (miRNA or miR), long-chain noncoding RNA (lncRNA) and circular RNA (circRNA) are currently the most studied ncRNAs with high therapeutic potential (He et al., 2020). It has been found that miR-21 can increase the activity of ERK-MAP kinase in fibroblasts, affect its structure and function, and control the degree of MIF (Thum et al., 2008). It has been reported that in the animal model of ischemia/reperfusion injury, inhibition of miR-21 can lead to the reduction of MIF and cardiac hypertrophy, and the overall improvement of cardiac function (Hinkel et al., 2020). In addition, in pressure overload induced cardiac hypertrophy rats, miR-1 can reduce cardiac fibrosis and reverse cardiac hypertrophy by targeting Fibulin-2 (Fbln2), a protein involved in extracellular matrix remodeling. The cardioprotective effect of miR-1 is the result of extracellular matrix remodeling, the improvement of calcium treatment and the inactivation of mitogen activated protein kinase (MAPK) (Karakikes et al., 2013). It has also been proved that inhibition of miR-221 and miR-222 can lead to increased fibrosis and left ventricular dilatation in a pressure overload induced hypertrophic mouse model. MiR-221/222 family inhibits myocardial fibrosis and plays a cardioprotective role by down-regulating JUN N-terminal kinases 1 (JNK1), a kind of regulator of TGF-β signal pathway (Verjans et al., 2018). Although ncRNA targeting therapy for MIF in pathological myocardial hypertrophy shows good therapeutic prospects, there are still many limitations in its real use in clinical practice. For example, ncRNA can target different signal pathways in different organs at the same time, which is easy to produce adverse off target effects.

Epigenetic modification refers to dynamic and reversible changes in chromatin accessibility and post-translational modification of histone tails without changing nucleotide sequences (Prinjha et al., 2012). Histone acetylation is the earliest and most deeply studied epigenetic modification, which plays an important role in chromatin remodeling and transcriptional regulation (Scher et al., 2007). Histone acetylation regulated gene transcription relies on “epigenetic readers”, that is, acetyl-binding proteins that recognize acetylated lysine in histones and recruit transcription regulatory complexes to chromatin (Wang et al., 2021). Almost all “epigenetic readers” contain bromodomains - about 110 amino acid modules that exist in many chromatin related proteins (Scher et al., 2007). The bromodomain and extra terminal domain (BET) family is a subfamily of bromodomain containing proteins (BRDs), which is composed of four proteins (BRD2, BRD3, BRD4 and BRDT) (Gyuris et al., 2009). Therefore, epigenetic modifications and protein translation can be regulated by affecting the BET family, and protein translation regulation may inhibit the transdifferentiation of fibroblasts into myofibroblasts through transcriptional elongation or inhibition of SMADs signaling. Studies have shown that in the mouse model of cardiac hypertrophy induced by aortic coarctation, BRD4 down-regulation can reduce collagen accumulation and inhibit myocardial fibrosis by reducing the production of reactive oxygen species (ROS) (Zhu et al., 2020, 4). In cardiac fibroblasts, TGF-β1 leads to the production of ROS that regulates the transdifferentiation of myofibroblasts. The significant role of ROS in fibrosis can be initiated by TGF-β1/SMADs signaling (Rhyu et al., 2005). BRD4 inhibition can reduce the expression of TGF-β1 and p-SMAD2/3 triggered by Ang II, thereby reducing ROS production to inhibit cardiac fibrosis and relieve cardiac hypertrophy (Zhu et al., 2020, 4). It has also been reported that BRD2 knockout can reverse cardiac hypertrophy, cardiac fibrosis and cardiac dysfunction in rats with pathological cardiac hypertrophy induced by isoprenaline (Lin et al., 2022). The mechanism may be related to BRD2 regulating the expression of citrate cycle gene, but the specific process is not clear. Therefore, BET family may be a new therapeutic target against MIF in pathological myocardial hypertrophy.

ER is an organelle involved in protein folding, lipid biosynthesis and calcium homeostasis (Kaufman, 1999). ER stress refers to changes in the intracellular environment that interrupt protein processing, lead to the accumulation of unfolded proteins in the endoplasmic reticulum and activate the unfolded protein response (UPR) (Ron and Walter, 2007). Although the causes of cardiac hypertrophy and fibrosis are diverse, they may all be expressed in the form of ER stress response in a common cellular mechanism (Luo et al., 2015a). At present, the mechanism of ER stress regulating myocardial hypertrophy and interstitial fibrosis is not clear, but some people have proposed that it may be related to involvement of CaN-MEF2c signaling pathways in cardiomyocytes and TGFβ1-SMAD2/3 signaling pathways in cardiac fibroblasts (Zhang et al., 2010; Luo et al., 2015b). Studies have shown that 4-phenylbutyric acid (4-PBA), an inhibitor of ER stress, can prevent myocardial hypertrophy and interstitial fibrosis caused by pressure overload by reducing ER stress (Luo et al., 2015a). 4-PBA inhibits ER stress by directly targeting mutant and/or misfolded proteins in cells and assisting appropriate protein transport to eliminate intracellular UPR (Gonzales et al., 2015). In addition, stimulator of interferon genes (STING), which mainly exists in ER, has also been shown to affect myocardial hypertrophy and fibrosis. In aortic coarctation induced cardiac hypertrophy mice, STING knockout in cardiomyocytes inhibited ER stress and the release of inflammatory factors, thereby inhibiting the fibrosis of cardiac fibroblasts (Zhang et al., 2020). All these indicate that inhibiting ER stress may be an effective treatment strategy to improve MIF in pathological cardiac hypertrophy.

Recently, the fundamental role of mitochondrial dysfunction in fibrosis has noticeably attracted clinicians’ attention. Cardiomyocytes are obligatory aerobic cells, and their survival depends on oxidative metabolism and stable demand for energy, which is provided by their mitochondria. Mitochondrial dysfunction is mainly manifested in mitochondrial structure and DNA damage, as well as changes in cellular oxidative protein activity (Li et al., 2020). Mitochondria are the main source of reactive oxygen species (ROS), a byproduct of oxygen metabolism. When mitochondria are dysfunctional, Ca^2+^ overload in mitochondria will lead to the induction of oxidative stress and the loss of ATP synthesis, resulting in an imbalance between biogenesis and the elimination of reactive oxygen species. When the production rate of ROS exceeds the detoxification rate of endogenous antioxidant defense, the permeability transition pore of mitochondrial inner membrane opens, which makes solute enter passively and leads to mitochondrial osmotic swelling (Weber et al., 2013). Eventually, these basic organelles degenerate and cardiomyocytes undergo necrosis, followed by fibrosis. This mechanism of cardiomyocyte necrosis “mitochondriocentric signal-transducer-effector “pathway. One strategy to reduce myocardial fibrosis is an intervention targeting mitochondria to prevent cardiomyocyte necrosis. The aim of this mitochondrial targeted cardiac protection strategy is to block the necrosis caused by the mitochondrial central signal transduction pathway by preventing mitochondrial Ca^2+^-overload under the sarcolemma. This includes the use of targeted antioxidants or inhibition of the opening of mitochondrial intimal permeability transition pores (Dai et al., 2011; Asemu et al., 2013; Segura et al., 2014). Such strategies include nutrients (flavonoids), drugs (cyclosporin A or third-generation *ß*-adrenoreceptor antagonists), inhaled hydrogen or expressed microRNAs (Bomb et al., 2016). For instance, some experiments have shown that in mice with ischemia-reperfusion injury, miRNA-214 regulates cardiomyocyte Ca^2+^ by inhibiting Na^2+^/Ca^2+^ exchange, preventing cardiomyocyte necrosis caused by cytosol and mitochondrial Ca^2+^ overload, so as to play a cardiac protective role (Aurora et al., 2012). In addition, it has also been proposed that miR-574 regulates the expression of family with sequence similarity 210 member A (FAM210A)and antagonizes cardiac fibrosis in mice with cardiac hypertrophy caused by aortic constriction. FAM210A acts as a novel regulator of the expression of mitochondrial encoded proteins, while miR-574 restricts the expression of FAM210A and may act as a molecular brake to maintain mitochondrial homeostasis and normal cardiac function (Wu et al., 2021).

## 6 Conclusion

MIF can be accompanied by pathological myocardial hypertrophy caused by various heart diseases, which can be induced by different stimuli in different heart diseases. In patients with pathological myocardial hypertrophy, due to different causes, the specific manifestations and characteristics of MIF are also different. And in patients with pathological myocardial hypertrophy, MIF affects their outcome and prognosis, so the evaluation of the etiology and degree of MIF should be paid attention to and are worthy of further investigation. Accordingly, the diagnosis and treatment of MIF in pathological myocardial hypertrophy should also be based on disease specificity and case-specific analysis, so as to provide individualized management for patients with pathological myocardial hypertrophy. Moreover, CMR imaging and circulating biomarkers should be reasonably used to ensure the accuracy and precision. Finally, although several anti-MIF strategies have been proposed, most of these strategies are idealized and difficult to implement in clinical practice, thus, the transformation from theory to practice still needs to be developed.
